# Body Mass Index and Its Role in Total Laparoscopic Hysterectomy

**DOI:** 10.1155/2014/787604

**Published:** 2014-10-28

**Authors:** Shilpa Bhandari, Pallavi Agrawal, Aparna Singh

**Affiliations:** Department of Obstetrics and Gynaecology, Sri Aurobindo Institute of Medical Sciences, Indore Ujjain Highway, Indore 453111, India

## Abstract

*Objective.* To evaluate operative and perioperative outcomes in patients undergoing total laparoscopic hysterectomy according to their body mass index. *Method.* A retrospective study was performed for patients undergoing total laparoscopic hysterectomy at a tertiary care center for a period of 4 years. Patients were divided into two groups: obese (BMI > 30 Kg/m^2^) and nonobese (BMI < 30 Kg/m^2^). Duration of surgery, intraoperative blood loss, successful laparoscopic completion, and intraoperative complications were compared in two groups. *Result.* A total of 253 patients underwent total laparoscopic hysterectomy from January 2010 to December 2013. Out of them, 105 women (41.5%) had a BMI of more than 30 kg/m^2^. Overall, the mean blood loss was 85.79 ± 54.17 mL; the operative time was 54.17 ± 19.83 min. The surgery was completed laparoscopically in 244 (96.4%) women while laparotomy was done in 4 cases and vaginal suturing and closure of vault were done in 5 cases. Risk of vaginal assistance was higher in obese patients whereas out of the 4 conversions to laparotomy 3 had BMI < 30 kg/m^2^. The operative time was increased as the BMI of patient increased. *Conclusions.* Total laparoscopic hysterectomy is a safe and effective procedure for obese patients and can be performed with an efficacy similar to that in nonobese patients.

## 1. Introduction

Obesity and comorbidities associated with it are well known factors that negatively affect surgical outcomes. Since higher BMI is a predisposing factor for abnormal uterine bleeding, endometrial hyperplasia, adenomyosis, and so forth, many females of higher BMI may require hysterectomy. In the past, laparoscopy was technically considered challenging in obese patients and was often considered a relative contraindication. But with significant advances in laparoscopic techniques this has come under review [1]. Abdominal hysterectomy has shown to be associated with higher rates of complications like wound infection, pelvic abscess, and longer postoperative stay in obese than nonobese patients [2]. In the past, number of randomized controlled trials have compared abdominal hysterectomy with laparoscopic assisted vaginal hysterectomy (LAVH) and found it to be in favor of the latter in terms of complications, blood loss, operating time pain, and hospital stay [3–6]. Total laparoscopic hysterectomy is viable option to LAVH especially in obese patients where vaginal dissection of cervix and lower uterine segment may be difficult for want of space and exposure.

In present retrospective study patient demographics, indication for surgery, intraoperative data, and complications were recorded for duration of 4 years in a single surgeon's practice in a teaching hospital and were analyzed to evaluate surgical parameters in performing total laparoscopic hysterectomy by BMI categories. The main outcome measures were duration of surgery, intraoperative blood loss, number of complications, and duration of hospital stay.

## 2. Materials and Method

The study was performed over a 4-year interval (January 2010 to December 2013) and all cases were operated by simple total laparoscopic hysterectomy at a tertiary teaching hospital by a single surgeon. Cases with abnormal uterine bleeding, fibroid, adenomyosis, endometrial hyperplasia were included in study. Patients with history of more than 2 open abdominal surgeries and reports documenting severe abdominal or intestinal adhesions or overt malignancy were excluded from the study.

Body mass index was calculated by dividing a person's weight in kilograms by the square of their height in meters. No patient was refused laparoscopic approach solely on the basis of her BMI. Presence of cardiac comorbidity was not considered a contraindication of laparoscopic hysterectomy unless it contraindicated prolonged steep Trendelenburg position or use of pneumoperitoneum.

Total laparoscopic hysterectomy was defined as completing the entire surgical procedure including the closure of vagina laparoscopically. Uteri were delivered vaginally or by morcellation depending on their sizes. Need for closure of vault vaginally or use of vaginal route to complete any part of surgical procedure was documented separately. All cases were initiated with one 10 mm optic supraumbilical port and three 5 mm ancillary ports. Need for additional port was documented separately. Bipolar rotating forceps (ENDOPATH Bipolar Forceps, Johnson & Johnson, USA) and ultrasonic scissors (harmonic scalpel, Ethicon, Johnson & Johnson, USA) were used for coagulation and cutting. Vault was closed endoscopically by Vicryl number 1-0 (Ethicon, Johnson & Johnson, USA) by continuous suturing. Postoperative antibiotic and analgesic protocol was standardized for all cases. Oral intake was started with the resumption of bowel activity as observed on auscultation of abdomen.

Patient data was reviewed for age, height, weight, parity, preoperative diagnosis, procedural details, duration of procedure, blood loss during procedure, duration of hospital stay, postoperative fever, wound infection, vaginal discharge, and readmission. Main outcome measures were duration of surgery, hospital stay, blood loss, and complications.

Statistical analysis was done on SPSS 20.0. Fisher exact test was performed to see the difference in frequencies of categorical data in between two groups and Student's *t*-test was performed to see the difference in mean values of quantitative date.

## 3. Result

During the study period, 279 patients underwent attempted total laparoscopic hysterectomy: 8 patients were excluded as they were converted to abdominal hysterectomy after initial diagnostic laparoscopy. Of these, 3 were suspicious of malignancy, 4 were cases of frozen pelvis not amenable to laparoscopic surgery, and in 1 a large ovarian mass was seen which needed to be removed in toto. Seven cases in which hysterectomy was done along with a separate surgical procedure (appendectomy, cholecystectomy, and hernia repair) were excluded from the study. 11 cases were excluded from the study due to incomplete data. Total patients included in study were 253.

The mean age of 253 patients was 46.0 ± 5.1 years with a median parity of two. The mean BMI of the patients was 30.04 ± 5.88 kg/m^2^ (range 20.18 to 48.6). Out of 253 patients, 148 patients had BMI ≤ 30.0 kg/m^2^ (Group 1) and 105 had BMI > 35.0 kg/m^2^ (Group II).

The most common indication of surgery was abnormal uterine bleeding (39.7%) followed by fibroid, cervical dysplasia, endometrial hyperplasia, and chronic pelvic pain. Obese patients (BMI > 30 kg/m^2^) had more chances of having abnormal uterine bleeding and endometrial hyperplasia than nonobese patients.

244 cases (96.4%) were completed by using purely endoscopic technique whereas 4 were converted to laparotomy, while 5 required vaginal assistance in closure of vault. Out of the 5 cases requiring vaginal assistance, 3 had BMI more than 45. No urological injury or injury to any major vascular structure was seen in the series, whereas 1 case of bowel injury was noted intraoperatively. The clinical parameters are summarized in Table 1. There was no difference in incidence of major complications in between two groups (Table 2). Most of the minor complications did not result in prolongation of admission and were managed in the postoperative period in an outdoor basis. The incidence of minor complications was statistically significantly higher in obese groups as compared to nonobese groups (Table 2). Major complications recorded were intraoperative hemorrhage and bowel injury in 8 patients and 1 patient, respectively; minor complications recorded were intraoperative blood loss of more than 200 mL, postoperative temperature of >100°F on two or more occasions 6 hr apart, vaginal bleeding, and vaginal discharge (Table 3).

We found a significant correlation of BMI with the operative time (*r* = 0.247, *P* < 0.0001) and duration of hospital stay (*r* = 0.295, *P* < 0.0001). However, there was no correlation of BMI with the estimated blood loss during surgery (*r* = 0.074, *P* = 0.239).

Vaginal assistance for vault closure was more commonly required in obese patients as compared to nonobese ones (*P* value 0.012). The failure of insufflation and use of extra port were statistically not significant in between two groups (Table 4).

## 4. Discussion

The prevalence of obesity in this country and throughout the industrialized world is such that the practicing surgeon cannot reasonably expect to avert its many implications for patient care.

Previously, many authors have compared the relationship of BMI with outcomes in laparoscopic hysterectomy. A prospective study by Holub et al. [7] showed a nonsignificant trend toward an increased rate of major operative complications in a group of 54 obese patients undergoing laparoscopic hysterectomy. Only half of the patients in that study underwent attempts at total laparoscopic hysterectomy, whereas the remaining half were attempted as laparoscopically assisted vaginal hysterectomies, a procedure shown by Milad et al. [8] to be associated with greater morbidity than supracervical laparoscopic hysterectomy. Ostrzenski [9] showed no increased rate of complications in his series of total laparoscopic hysterectomies in 11 obese women. This was a pilot phase report in which obesity was defined as a function of ideal body weight, rather than BMI. In 2003, O'Hanlan et al. [10] reported on 330 patients, stratified according to BMI groups, who underwent total laparoscopic hysterectomy. Those retrospective studies included 78 obese women and found similar mean operating time, mean operative blood loss, mean length of hospital stay, and complication rates across all BMI groups.

Laparoscopy in obese patients can be technically challenging for the surgeon but is more rewarding for the patient. The major initial technical difficulties encountered with higher BMI are creation and maintenance of pneumoperitoneum. Veress needle entry, in our case series, was the standard method for creation of pneumoperitoneum in all BMI groups. Due to increasing skin thickness, counter traction with a skin fold is not possible in cases of higher BMI; vertically directed Veress needle entry yields satisfactory result. It is possible that in thin patients the Veress may tentatively be directed more towards the pubic symphysis for fear of injuring major vital structures below umbilicus. In patients of higher BMI, a longer Veress was used which may also have contributed to fewer cases of failed insufflation.

Maintenance of pneumoperitoneum is technically more difficult in patients of high BMI due to presence of concurrent cardiorespiratory compromise. The inspiratory pressures are more in women with higher BMI especially when in Trendelenburg position due to the weight of the abdominal walling, bowel, and omentum that reduces ventilator compliance during surgery. In the present case series, no cases were terminated prematurely due to ventilator issues; however, the steep Trendelenburg position was not used for patients with BMI more than 40.

Previous studies have shown an increase in conversion to laparotomy with BMI more than 30. In a review of 2,530 attempted gynecologic laparoscopic surgeries, Sokol et al. [11] determined that a BMI greater than 30 kg/m^2^ placed patients at a more than 2-fold risk of unintended laparotomy. Eltabbakh et al. [12] noted similar findings in a review of 47 obese patients who underwent operative gynecologic laparoscopies. In our case series, no conversion to laparotomy was attributed to BMI or reasons associated with it. Out of 253 cases, 4 were converted to laparotomy. Of these only 1 was obese. This was a case of adenomyosis with concurrent extensive endometriosis. The increased transverse diameter of the uterus rendered visualization difficult. In the remaining 3 cases, the BMI was less than 30. Of these 1 was suspected malignant ovarian mass, one had extensive abdominal adhesions rendering laparoscopy difficult, and in 1 bowel injury was noted for which laparotomy was done.

In present study, 8 patients were nulliparous whereas 64 patients had never had previous vaginal deliveries and 27 patients had 1 or more Cesareans along with vaginal deliveries. In such cases, the vaginal space is often inadequate and vaginal hysterectomy becomes difficult. In laparoscopic approach, the constraints of vagina are easily dealt with. Many gynecologists prefer vaginal hysterectomy especially in cases of mild descent (NDVH). In such cases too, we have found TLH to be an efficient approach especially as it allows better vaginal support with uterosacral ligaments which provides a visible elevation to the vaginal vault, which is not always feasible from below. In patients with higher BMI, the peripheral fat precludes good visualization of vagina and with increasing BMI the space at vaginal end becomes less. In these cases, laparoscopy becomes a much easier approach. Only a prospective randomized control trial can compare the safety and efficacy of TLH and NDVH in women irrespective of their vaginal capacities.

Longer operating time remains an important issue with patients of higher BMI. One of the largest, recent studies included 1460 patients who underwent laparoscopic hysterectomy (LH) for benign conditions and showed that a BMI > 30 kg/m^2^ was not associated with an increased risk of peri- or postoperative complications, but a longer operating time was found for the obese women [13]. In present study, operating time was found significantly correlated with BMI. Many of the previous studies have not noted a difference in operating time in their series.

In present series vaginal assistance was more frequently required in obese patients. This could account for the longer operating time for these groups. The operating time is expected to reduce with increase in experience and expertise.

Our complication rates compare favorably with open laparotomy data. With open laparotomy, obese patients have been shown to have a higher incidence of wound infection and other complications resulting in extended hospitalizations and additional procedures, directly proportional to the BMI. We observed a 13% total complication rate for our series. This rate is slightly higher than traditional transabdominal or more recent laparoscopic hysterectomy series. In a study done by Osler et al. [14], high BMI were associated with overall increased risk of complications mainly due to bleeding and infection. This risk was attributed more to patients undergoing abdominal hysterectomy rather than laparoscopic hysterectomy. In present study, we observed that secondary hemorrhage was higher in obese patient as compared to nonobese patients.

In practice, the number of patients in our study is relatively large. However, the observational nature of a retrospective study precludes absolute conclusions. Gynecologic surgeons having less operative exposure to obese patients or less experience with laparoscopy may experience higher rates of complications and conversion to laparotomy when attempting laparoscopic hysterectomy for obese women. These limitations should be considered when attempting to apply our findings to one's individual clinical practice. This comparison should be viewed more as a feasibility or pilot study, serving as an indicator of future research focus.

The Cochrane review on hysterectomy [15] recommends VH in preference to AH as a standard approach for smaller uteri <300 g or when feasible due to equal or significantly better outcomes on all parameters. However, the review does not consider whether the influence of other risk factors for complications such as BMI varies by route of surgery. Thus, our results support and append to the recommendation that TLH could be considered whenever possible even in patients of higher BMI. Despite limitations, our study adds to the growing body of literature suggesting that total laparoscopic hysterectomy can be performed safely for obese patients, with complication rates similar to those for nonobese patients.

In India, where 25% of the adult population is obese and where laparoscopy is rapidly becoming the standard of care, it becomes important to focus on the feasibility and safety of performing laparoscopic approaches on larger women. Our data demonstrate that a total laparoscopic hysterectomy is as feasible and safe for patients with high BMI as it is for patients of ideal BMI. Complications are minimized with training, experience, and a meticulous approach. On the basis of this cohort of cases, randomized prospective studies comparing total laparoscopic hysterectomy with TAH, vaginal hysterectomy, and NDVH should be performed, including women with the full spectrum of BMI to validate the utility of each procedure in the population.

## Figures and Tables

**Table 1:** Indications for surgery in Obese and Non Obese patients.

Diagnosis	Non-Obese (*n* = 148)	Obese (*n* = 105)
Fibroid	36 (24.3)	25 (23.8)
Adenomyosis	5 (3.4)	2 (1.9)
Abnormal uterine bleeding	58 (39.2)	43 (41.0)
Endometrial hyperplasia	14 (9.5)	4 (3.8)
Cervical dysplasia	12 (8.1)	9 (8.5)
Chronic cervicitis	8 (5.4)	7 (6.7)
Chronic pelvic pain	6 (4.1)	12 (11.4)
Ovarian mass	3 (2.0)	2 (1.9)
Tubo-ovarian mass	6 (4.1)	0 (0.0)
Ovarian cyst	0 (0.0)	1 (0.9)

**Table 2:** Clinical and observational parameters in two groups.

	Non Obese	Obese	*P* value
Duration of surgery (Minutes)	52.26 ± 20.75	56.86 ± 18.22	0.069
Estimated blood loss (mL)	83.61 ± 114.9	88.96 ± 62.7	0.672
Length of hospital stay (days)	1.67 ± 0.92	1.88 ± 0.89	0.076
Minor complication	10 (7.4)	22 (20.9)	0.001
Major complication	5 (3.4)	4 (3.8)	0.8552
Intra-operative hemorrhage	4 (2.7)	4 (3.8)	0.721
Bowel Injury	1	0	0.3987
Readmission	1 (0.7)	0 (0.0)	0.3987
Blood transfusion	4 (2.7)	1 (0.9)	0.5980

**Table 3:** Minor complications in two groups.

	Non Obese	Obese	*P* value
Post op fever	5 (3.4)	6 (5.7)	0.533
Vaginal discharge	5 (3.4)	8 (7.6%)	0.155
Vaginal cuff dehiscence	0 (0.0)	1 (0.95)	0.415
Port site infection	0 (0.0)	3 (2.9%)	0.070
Secondary hemorrhage	0 (0.0)	4 (3.8)	0.029

**Table 4:** Operative events in two groups.

	Non Obese	Obese	*P* value
Failed insufflation/extra peritoneal insufflation	4 (2.7)	3 (2.9)	1.000
Conversion to laparotomy	3 (2.0)	1 (0.95)	0.644
Extra port required	2 (1.4)	5 (4.8)	0.103
Vaginal closure of vault	0 (0.0)	5 (4.8)	0.012

## References

[1] Curet M. J. (2000). Special problems in laparoscopic surgery: previous abdominal surgery, obesity, and pregnancy. *Surgical Clinics of North America*.

[2] Goodman M. T., Hankin J. H., Wilkens L. R. (1997). Diet, body size, physical activity, and the risk of endometrial cancer. *Cancer Research*.

[3] Kung F.-T., Hwang F.-R., Lin H., Tai M.-C., Hsieh C.-H., Chang S.-Y. (1996). Comparison of laparoscopically assisted vaginal hysterectomy and abdominal hysterectomy in Taiwan. *Journal of the Formosan Medical Association*.

[4] Polet R., de Jong P., van der Spuy Z. M., Shelton M. (1996). Laparoscopically assisted vaginal hysterectomy (LAVH)—an alternative to total abdominal hysterectomy. *South African Medical Journal*.

[5] Schneider A., Merker A., Martin C., Michels W., Krause N. (1997). Laparoscopically assisted vaginal hysterectomy as an alternative to abdominal hysterectomy in patients with fibroids. *Archives of Gynecology and Obstetrics*.

[6] Malur S., Possover M., Michels W., Schneider A. (2001). Laparoscopic-assisted vaginal versus abdominal surgery in patients with endometrial cancer—a prospective randomized trial. *Gynecologic Oncology*.

[7] Holub Z., Jabor A., Kliment L., Voráček J., Lukác J. (2000). Comparison of two procedures for laparovaginal hysterectomy: a randomized trial. *European Journal of Obstetrics Gynecology and Reproductive Biology*.

[8] Milad M. P., Morrison K., Sokol A., Miller D., Kirkpatrick L. (2001). A comparison of laparoscopic supracervical hysterectomy vs laparoscopically assisted vaginal hysterectomy. *Surgical Endoscopy*.

[9] Ostrzenski A. (1999). Laparoscopic total abdominal hysterectomy in morbidly obese women: a pilot-phase report. *Journal of Reproductive Medicine for the Obstetrician and Gynecologist*.

[10] O'Hanlan K. A., Lopez L., Dibble S. L., Garnier A.-C., Huang G. S., Leuchtenberger M. (2003). Total laparoscopic hysterectomy: body mass index and outcomes. *Obstetrics & Gynecology*.

[11] Sokol A. I., Chuang K., Milad M. P. (2003). Risk factors for conversion to laparotomy during gynecologic laparoscopy. *Journal of the American Association of Gynecologic Laparoscopists*.

[12] Eltabbakh G. H., Piver M. S., Hempling R. E., Recio F. O. (1999). Laparoscopic surgery in obese women. *Obstetrics and Gynecology*.

[13] Chopin N., Malaret J. M., Lafay-Pillet M.-C., Fotso A., Foulot H., Chapron C. (2009). Total laparoscopic hysterectomy for benign uterine pathologies: obesity does not increase the risk of complications. *Human Reproduction*.

[14] Osler M., Daugbjerg S., Frederiksen B. L., Ottesen B. (2011). Body mass and risk of complications after hysterectomy on benign indications. *Human Reproduction*.

[15] Nieboer T. E., Johnson N., Lethaby A. (2009). Surgical approach to hysterectomy for benign gynaecological disease. *Cochrane Database of Systematic Reviews*.

